# Non-invasive high-frequency oscillatory ventilation versus nasal continuous positive airway pressure in preterm infants with respiratory distress syndrome: Study protocol for a multi-center prospective randomized controlled trial

**DOI:** 10.1186/s13063-018-2673-9

**Published:** 2018-06-14

**Authors:** Xing-Wang Zhu, Yuan Shi, Li-Ping Shi, Ling Liu, Jiang Xue, Rangasamy Ramanathan, for the NHFOV Study Group, Xing-Wang Zhu, Yuan Shi, Lei Bao, Cheng-Jun Liu, Li-Ping Shi, Jiang Xue, Rong Ju, Ling Liu, Xi-Rong Gao, Xiao-Yun Zhong, Hui-Qing Sun, Yang-Fang Li, Kun Du, Hong Cao, Zhao-qing Yin, Ming Yi, Jun Yan, Zhan-Kui Li, Wei-Wei Gao, Rui Cheng, Lai-Shuan Wang, Vladimiras Chijenas

**Affiliations:** 1 Daping Hospital, Research Institute of Surgery, Third Military Medical University, Chongqing, 400042 China; 2 Jiulongpo People’s Hospital, Chongqing, 400024 China; 3 The Children’s Hospital of Zhejiang University School of Medicine, Hangzhou, 310000 China; 4 The Second Hospital of Shandong University, Jinan, 250000 China; 5 Guiyang Maternity and Child Health Care Hospital, Guiyang, 550000 China; 6 LAC+USC Medical Center, Keck School of Medicine, University of Southern California, Los Angeles, CA USA; 7 Division of Neonatology, Department of Pediatrics, LAC+USC Medical Center, Keck School of Medicine of University of Southern California, Los Angeles, CA 90033 USA

**Keywords:** Non-invasive high-frequency oscillatory ventilation, Nasal continuous positive airway pressure, Respiratory distress syndrome, Preterm infants, Surfactant, Invasive mechanical ventilation

## Abstract

**Background:**

Invasive mechanical ventilation (IMV) is associated with the development of adverse pulmonary and non-pulmonary outcomes in very premature infants. Various modes of non-invasive respiratory support are increasingly being used to decrease the incidence of bronchopulmonary dysplasia. The aim of this trial is to compare the effect of non-invasive high-frequency oscillatory ventilation (NHFOV) and nasal continuous positive airway pressure (NCPAP) in preterm infants with respiratory distress syndrome (RDS) as a primary non-invasive ventilation support mode.

**Methods/design:**

In this multi-center randomized controlled trial, 300 preterm infants born at a gestational age of 26^6/7^ to 33^6/7^ weeks with a diagnosis of RDS will be randomized to NHFOV or NCPAP as a primary mode of non-invasive respiratory support. The study will be conducted in 18 tertiary neonatal intensive care units in China.

The primary outcome is the need for IMV during the first 7 days after enrollment in preterm infants randomized to the two groups. The prespecified secondary outcomes include days of hospitalization, days on non-invasive respiratory support, days on IMV, days on supplemental oxygen, mortality, need for a surfactant, severe retinopathy of prematurity requiring laser treatment or surgery, patent ductus arteriosus needing ligation, bronchopulmonary dysplasia, abdominal distention, air leak syndromes, intraventricular hemorrhage (≥ grade 3), spontaneous intestinal perforation, necrotizing enterocolitis (≥II stage), and nasal trauma. Other secondary outcomes include Bayley Scales of Infant Development at 18–24 months of corrected age.

**Discussion:**

In recent decades, several observational studies have compared the effects of NHFOV and NCPAP in neonates as a rescue mode or during weaning from IMV. To our knowledge, this will be the first multi-center prospective, randomized controlled trial to evaluate NHFOV as a primary mode in preterm infants with RDS in China or any other part of the world. Our trial may help to establish guidelines for NHFOV in preterm infants with RDS to minimize the need for IMV, and to decrease the significant pulmonary and non-pulmonary morbidities associated with IMV.

**Trial registration:**

ClinicalTrials.gov, NCT03099694. Registered on 4 April 2017.

**Electronic supplementary material:**

The online version of this article (10.1186/s13063-018-2673-9) contains supplementary material, which is available to authorized users.

## Background

Respiratory distress syndrome (RDS) due to surfactant deficiency is the leading cause of respiratory failure in preterm infants [1]. Early non-invasive positive pressure ventilation has become a recommended strategy for the respiratory management of preterm infants with RDS [2]. In addition to nasal continuous positive airway pressure (NCPAP), various types of non-invasive ventilation (NIV) modes have been used in the treatment of RDS, including heated humidified high-flow nasal cannula, biphasic NCPAP, and nasal intermittent positive airway pressure [3]. However, clinical trials have shown that 25 to 67% of very low birth weight preterm infants fail the above-mentioned NIV modes and require invasive mechanical ventilation (IMV) [4, 5]. To minimize the need for IMV, non-invasive high-frequency oscillatory ventilation (NHFOV) has been studied as a rescue treatment after failure of other NIV modes or following extubation from IMV during the weaning phase [6]. However, to date, no studies have been published on the efficacy of NHFOV as a primary mode of respiratory support in preterm infants [7]. In the present trial, we aim to compare the effect of NHFOV and NCPAP in preterm infants with RDS as a primary NIV mode. Our main hypothesis is that NHFOV is more effective in the treatment of preterm infants with RDS than NCPAP when used as a primary NIV mode.

## Methods/design

### Aim

The primary aim of this trial is to compare the need for IMV during the first 7 days of life in infants randomized to NHFOV vs. NCPAP.

### Study design

**Fig. 1:**
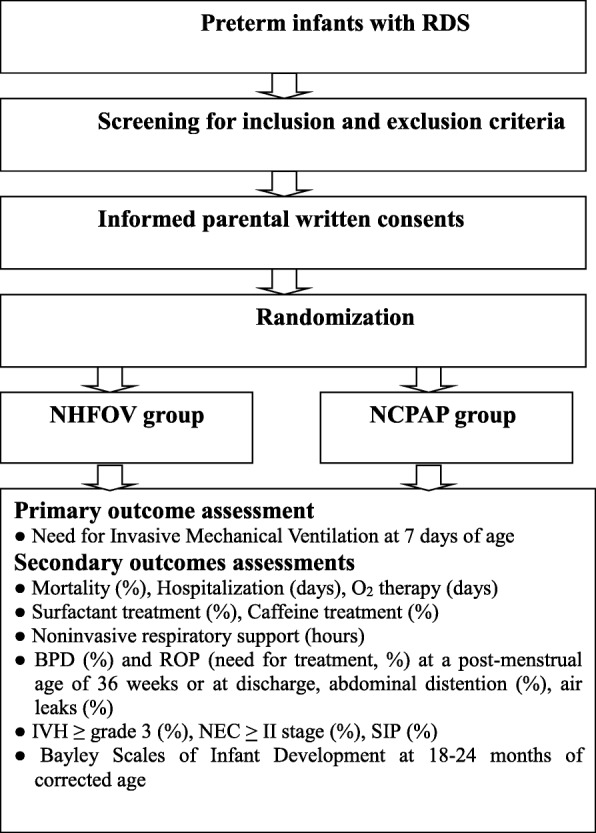
Flow diagram of the study protocol

This will be a multi-center prospective randomized controlled trial conducted in 18 tertiary neonatal intensive care units (NICUs) in China from May 2017 to July 2018. The schedule of trial enrollment, interventions, and assessments is presented in Additional file 1 (SPIRIT checklist). The trial will be performed in accordance with the prospective trial flow (Fig. 1) (SPIRITchecklist).

### Inclusion criteria

Children will be included if they meet all of the following inclusion criteria:

1. Their gestational age (GA) is between 26^0/7^ and 33^6/7^ weeks. GA will be determined by dates or a dating ultrasound. A Ballard examination will be performed. If the dates estimated by the Ballard examination are within 2 weeks of the obstetric estimate, the obstetric estimates will be used. If the dates are unknown or the examination and obstetric estimate differ by more than 2 weeks, the GA estimated by the Ballard examination will be used.
2. They have a diagnosis of RDS. The diagnosis of RDS will be based on clinical manifestations (tachypnea, nasal flaring, and or grunting) or RDS Silverman score >5.
3. Informed parental consent has been obtained.

### Exclusion criteria

Children will be excluded if they meet any of the following inclusion criteria:

1. Any baby intubated for resuscitation or for other reasons.
2. Major congenital malformations or known complex congenital heart disease.
3. Pulmonary hemorrhage.
4. Cardiopulmonary arrest needing prolonged resuscitation.
5. Transferred out of the NICU before randomization.

### Setting

We plan to enroll preterm infants born between 26^0/7^ and 33^6/7^ weeks of GA from 18 tertiary NICUs in China. These 18 tertiary NICUs have more than 800 NICU beds and annual admissions of nearly 8000 preterm infants with RDS each year. The first (XZ) and last authors (RR) take responsibility for the accuracy and completeness of the trial.

### Randomization

Intervention assignments will use sequentially numbered sealed, opaque envelopes after verification of eligibility. Infants will be stratified according to GA: 26^0/7^ to 27^6/7^ weeks, 28^0/7^ to 30^6/7^ weeks, 30^0/7^ to 31^6/7^ weeks, and 32^0/7^ to 33^6/7^ weeks. This procedure will be repeated for each group of four infants. A clinician who is not in the trial group will open the envelope and randomize patients. Infants born from multiple gestations will be assigned by individual randomization.

### Trial intervention

All subjects will be randomly allocated to either NHFOV or NCPAP after birth. NHFOV will be provided by a high-frequency ventilator (CNO, Medin, Germany or SLE5000, UK) via binasal prongs. All participating centers have expertise with using these high-frequency oscillatory ventilators. Guidelines for initial and maximum settings will be provided to all sites. Initial NHFOV settings will be a mean airway pressure of 6 cm H_2_O (range 6–10), which is equivalent to that for the NCPAP group and a frequency of 8 Hz (range 8–12). The amplitude produced by the Medin CNO device can be adjusted between 1 and 10, and the initial setting for the amplitude will be 7 (range 7–10). For the SLE5000, the initial settings will be the same as those for the Medin CNO device, except the inspiratory time will be 50% (1:1) and the amplitude will be 20 cm H_2_O (range 20–35 cm H_2_O).

Infants assigned to the NCPAP group will be started on a pressure of 6 cm H_2_O (range 6–8 cm H_2_O) by the continuous positive airway pressure system (CNO Medin, Germany, SLE5000, UK, or Carefusion, USA).

To minimize abdominal distension, an oro-gastric tube will be placed in the stomach and gas will be periodically aspirated during the study period in both groups. Short binasal prongs will be used in both groups and they will be changed periodically to reduce the risk of nasal injury. The best fitting ones will be used (the largest that fits the nares without blanching the surrounding tissues), based on the diameter of the nares. The manufacturer’s recommendations will be followed. Pacifiers will be used whenever possible to decrease air leaks from the mouth. No crossover will be allowed.

FiO_2_ will be adjusted to target oxygen saturation (SpO_2_) from 89% to 93% in preterm infants <30 weeks GA and from 90 to 94% in infants ≥30 weeks GA by pulse oximeter [8].

Surfactant (Poractant alfa, Chiesi Pharmaceuticals, Parma, Italy) at a dose of 200 mg/kg will be administered via the INSURE method (intubation, surfactant, and extubation) if an infant presents with the following: ≤30 weeks GA when FiO_2_ requirement >0.30 or >30 weeks GA when FiO_2_ requirement >0.40 [2]. In the INSURE technique, surfactant is administered via an endotracheal tube and after a brief period of positive pressure ventilation, patients will be extubated via the assigned mode. Infants undergoing the INSURE method will not be considered as having received IMV. Additional doses of surfactant may be given using the INSURE technique at the discretion of the clinician.

A caffeine citrate injection (Chiesi Pharmaceuticals, Parma, Italy) will be administered when infants present with moderate apnea (defined as three or more episodes in 24 h or a single episode requiring resuscitation and bag and mask ventilation). The initial loading dose is 20 mg/kg, and the maintenance dose is 5 mg/kg per day.

The criteria for intubation and IMV will be as follows [9, 10]: severe respiratory acidosis (P_a_CO_2_ > 65 mmHg with pH < 7.20), severe apnea and bradycardia (defined as recurrent apnea with >3 episodes per hour associated with heart rate <100/min or a single episode of apnea that requires bag and mask ventilation), hypoxemia (FiO_2_ > 0.5 with PaO_2_ < 50 mmHg from an arterial blood gas sample), severe respiratory distress, pulmonary hemorrhage, and cardiopulmonary arrest needing chest compressions.

The criteria for weaning from non-invasive respiration will be: (1) minimal or no signs of respiratory distress, (2) NHFOV mean airway pressure or NCPAP pressure <6 cm H_2_O, and (3) FiO_2_ <0.25 to achieve target SpO_2_.

### Outcomes

The primary outcome of this trial will be to determine the need for IMV in the first 7 days of life in preterm infants randomized to the two groups. Secondary outcomes include days of hospitalization, days on NIV, days on supplemental oxygen, pre-discharge mortality, surfactant doses, patent ductus arteriosus needing surgical ligation, stage III retinopathy of prematurity, bronchopulmonary dysplasia (BPD), abdominal distention, feeding intolerance, time to full feed, air leaks (including pneumothorax, pneumomediastinum, and pneumopericardium), intraventricular hemorrhage ≥ grade 3, spontaneous intestinal perforation, necrotizing enterocolitis, and presence of thick secretions causing an airway obstruction. Secondary outcomes also include Bayley Scales of Infant Development (Bayley III using MDI (Mental Developmental Index) and PDI (Psychomotor Developmental Index) scores) at 18–24 months of corrected age. BPD will be classified according to the National Institutes of Health consensus definition as mild, moderate, or severe [11]. A intraventricular hemorrhage will be classified following Papile et al. [12] and for necrotizing enterocolitis, Bell staging will be used [13].

### Data collection

Patient demographic data include: sex, birth weight, GA, Apgar score, mode of delivery, prenatal corticosteroid use (classified as complete if the mother received two doses of betamethasone or partial if less than two doses), premature rupture of the membrane before the onset of labor, RDS Silverman score, and clinical risk index for babies scores (CRIB-II; used to compare illness severity between the groups). Population characteristics will be used to compare outcomes between the two groups.

#### Clinical data

In addition to the need for IMV, the following clinical data will be collected: days of hospitalization, days on non-invasive respiratory support, mean airway pressure in both groups, days on supplemental oxygen, mortality, the need for surfactant, abdominal distention, air leaks, patent ductus arteriosus needing surgery, intraventricular hemorrhage ≥ grade III, spontaneous intestinal perforation, and necrotizing enterocolitis ≥ stage II.

#### Follow-up data

**Fig. 2:**
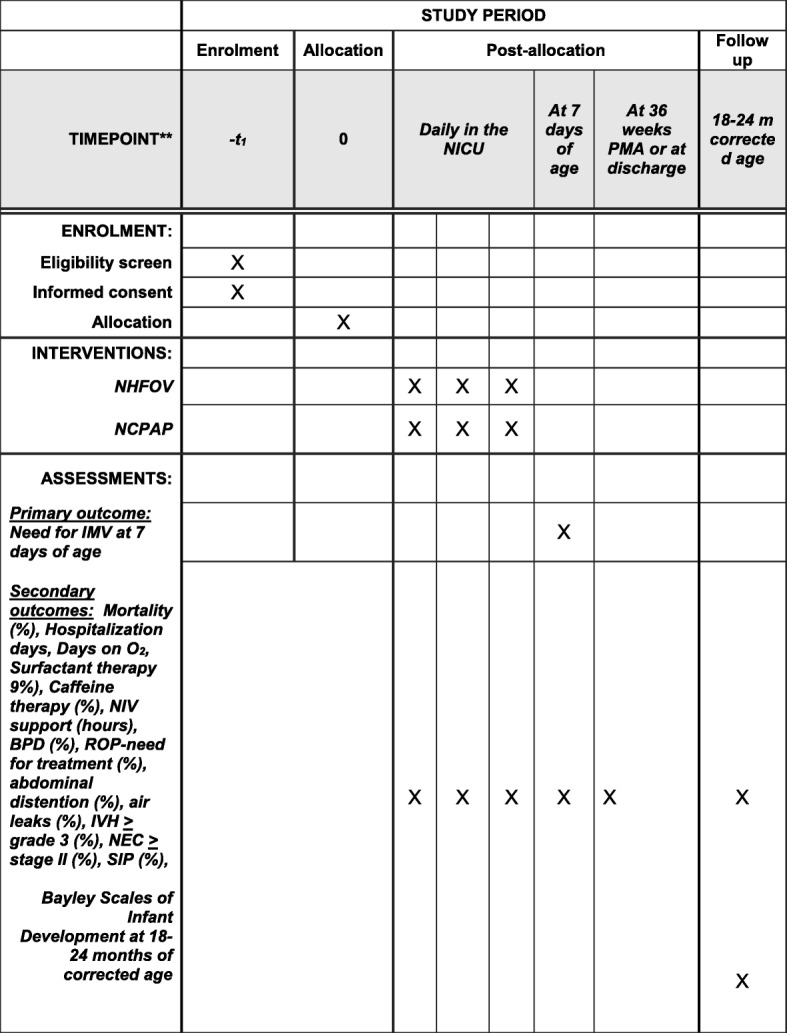
Follow-up schedule

Follow-up data will include the incidence of BPD and retinopathy of prematurity at a post-menstrual age of 36 weeks or at discharge, and Bayley Scales of Infant Development at 18–24 months of corrected age (Fig. 2).

### Sample size calculation

 The sample size estimation was calculated using PASS software. According to previous studies [4, 5], around 40% of preterm infants treated with early NCPAP and surfactant for RDS needed IMV. It is difficult to calculate a sample size for a study like ours, since this is the first study to investigate NHFOV vs. NCPAP as a primary support mode in preterm infants with RDS. For the primary outcome of the need for IMV, sample size was calculated based on the only study previously reported by Zhu et al. [14]. This trial showed that the need for IMV was significantly lower in the NHFOV compared with the NCPAP group (24.3% vs. 56.4%). Considering an alpha error rate of 0.05 and a power of 90%, 150 neonates will need to be enrolled in each arm (with a 1:1 design) to detect the same difference. We, therefore, plan to recruit at least 170 infants in each group, to account for dropouts. We have enrolled 300 patients as of January 2018. We anticipate completing enrollment by May 2018. Use of a relatively scale with Medin device is a minor study limitation.

### Statistical methods

Data will be analyzed using SPSS version 19. The statistical analyses include Student’s *t*-test for continuous data. Proportions will be compared using a chi-squared test. Fisher‘s exact test will be used for categorical data. The two predefined subgroups are 26^0/7–^29^6/7^weeks and 30^0/7–^33^6/7^ weeks GA, and subgroup analyses will be conducted for the primary outcome in the preterm infants. To evaluate further the effect of NHFOV on intubation within each subgroup, the test of the treatment-by-GA subgroup interaction will also be done using a paired binary logistic regression. We will include center as a variable in our multivariate analysis. For the preterm infants lost to follow-up, missing values for the primary and secondary outcomes will be replaced using multiple imputations. *P* < 0.05 will be regarded as statistically significant.

### Data safety monitoring board

The board will have the following members:

- Dr. Kris Sekar, Professor of Pediatrics, Oklahoma University Medical Center, Children’s Hospital, Oklahoma City, OK
- Dr. Jatinder Bhatia, Professor of Pediatrics, Medical College of Georgia, Augusta University, Augusta, GA
- Dr. Rowena Cayabyab, MD, MPH (Biostatistics and Epidemiology) Assistant Professor of Pediatrics, Keck School of Medicine of the University of Southern California, Los Angeles, CA

Dr. Cayabyab will also serve as a consultant for the statistical analysis.

### Registration number

The trial has been approved by the ethics committee of Daping Hospital, Research Institute of Surgery of the Third Military Medical University and registered at (NCT03099694).

## Discussion

In recent years, several clinical trials have compared the effects of NHFOV and NCPAP in neonates as a rescue mode or during weaning from IMV. These trials demonstrated that NHFOV applied a with nasopharyngeal tube is more beneficial than NCPAP in reducing CO_2_ levels [15–19]. Recently, two retrospective case series also reported that NHFOV could be applied in preterm infants as a rescue treatment after the failure of other NIV modes [15, 20]. However, there are some limitations in these trials: (1) small sample size, (2) lack of a prospective randomized trial design, and (3) the wide range of NHFOV parameters used in these trials. Given these limitations, a multi-center prospective randomized controlled trial is necessary to give a better evaluation of NHFOV as a primary mode of non-invasive support. To our knowledge, this will be the first multi-center prospective randomized controlled trial to evaluate of NHFOV as a primary non-invasive mode in preterm infants with RDS in China or any other part of the world. Our trial may help to establish guidelines for NHFOV in preterm infants with RDS to minimize the need for IMV, and to decrease the significant pulmonary and non-pulmonary morbidities associated with IMV. Some of the limitations in our study include: not setting an age limit at randomization, and not defining RDS severity other than using clinical findings and Silverman’s scoring,

### Trial status

At the time of this manuscript submission, enrollment is ongoing.

## Additional file

Additional file 1: SPIRIT Checklist, with details of study procedures and follow-up. (DOC 118 kb)

## Abbreviations

BPD: Bronchopulmonary dysplasia
GA: Gestational age
IMV: Invasive mechanical ventilation
NCPAP: Nasal continuous positive airway pressure
NHFOV: Non-invasive high frequency oscillatory ventilation
NICU: Neonatal intensive care unit
NIV: Non-invasive ventilation
RDS: Respiratory distress syndrome
